# A Rare Case of Metastatic Adrenal Melanoma

**DOI:** 10.7759/cureus.64182

**Published:** 2024-07-09

**Authors:** Rohma R Khan, Sunny Kahlon, Jason-Paul Maragh, Alyssa Kimble, Shanu Gupta

**Affiliations:** 1 Internal Medicine, University of South Florida, Tampa, USA

**Keywords:** multidisciplinary tumor board, flank pain, large adrenal mass, metastatic skin cancer, adult hospital medicine, immunotherapy-related adverse events, combined nivolumab and ipilimumab therapy, pheochromocytoma differential, adrenal melanoma, metastatic melanoma treatment

## Abstract

Metastatic melanoma presents a significant clinical challenge, characterized by its aggressive nature and propensity to spread to multiple organ systems. Despite advances in detection and treatment, managing metastatic disease remains complex. Here, we present the case of a 43-year-old male with metastatic melanoma displaying an unusual pattern of involvement, affecting the adrenal gland, liver, spleen, and bones. The diagnostic process was intricate, involving atypical hormonal profiles and a negative BRAF status, necessitating a comprehensive approach for accurate characterization and treatment selection. Immunotherapy demonstrated efficacy but also highlighted the emergence of immune-related adverse events, notably hyperglycemia. This case discusses the heterogeneous nature of metastatic melanoma and underscores the importance of a multidisciplinary approach, close monitoring, and consideration of evolving treatment strategies in its management.

## Introduction

Metastatic melanoma represents a severe and aggressive form of skin cancer known for its potential to spread widely and involve multiple organ systems. The incidence of melanoma has been on the rise, with about 100,000 cases expected to be diagnosed in 2024 [1]. Despite advancements in early detection and therapeutic modalities, a significant proportion of patients progress to develop metastatic disease [1]. The skin, liver, lungs, and bone are frequent sites of metastases in patients with advanced melanoma [2]. Previous case reports have highlighted the heterogeneous presentation and clinical course of metastatic melanoma involving multiple organs, but the concurrent involvement of the adrenal gland, liver, spleen, and bones remains rare in the literature [3,4]. In this case report, we present a comprehensive account of a patient diagnosed with metastatic melanoma manifesting as an adrenal mass along with liver, spleen, and osseous metastases. We aim to discuss the clinical presentation, radiological findings, pathological correlations, and therapeutic considerations, contributing to a deeper understanding of this rare metastatic pattern. 

## Case presentation

A 43-year-old male with a past medical history of hypertension and type 2 diabetes mellitus presented to an outside hospital for evaluation of bilateral flank pain accompanied by nausea, as well as pleuritic chest pain. He was afebrile, tachycardic to the 110s, and normotensive (124/79) on arrival. Initial labs were remarkable for elevated alkaline phosphatase at 134 U/L (normal range 38-113 U/L), normocytic anemia with hemoglobin of 11.3 g/dL (normal range 13-17 g/dL), and thrombocytosis with platelet count of 467 k/uL (normal range 150-400 k/uL). Computed tomography (CT) pulmonary embolism protocol was negative for pulmonary embolism. A CT of the abdomen/pelvis was obtained and revealed an 8.2 x 10 cm lesion in the left adrenal gland suspicious for malignancy. A CT scan completed nine months earlier showed a 5.5 cm left adrenal mass suspicious for underlying neoplasm; however, the patient was lost to follow-up.

**Figure 1:**
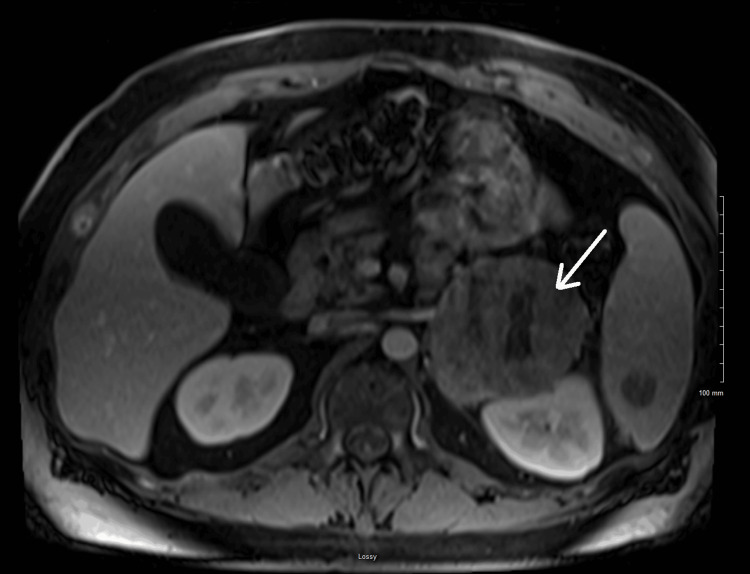
MRI image of the left adrenal mass MRI: Magnetic resonance imaging

At this presentation, additional lesions were identified on CT in the liver, spleen, right 12th rib, T5 vertebral body, and T10 vertebral body, raising concerns for metastasis from a primary adrenal malignancy. The patient denied any personal history of malignancy; his family history was notable for gastric cancer in his mother, diagnosed at the age of 60. He had no known occupational exposures; he denied alcohol and tobacco use. Magnetic resonance imaging (MRI) of the abdomen and a nuclear bone scan confirmed a left adrenal lesion measuring 12.2 cm in diameter (Figure 1) in addition to multiple osseous lesions. A hormonal work-up was initiated. While results were in progress, the patient was started on an alpha blocker (prazosin) in anticipation of the need for a biopsy or adrenalectomy. Labs returned revealing normal 24-hour urine epinephrine and norepinephrine, elevated 24-hour urine dopamine at 720 ug/d (normal range 71-485 ug/d), increased urine dopamine to creatinine ratio at 317 ug/g (normal range 0-250 ug/g), suppressed cortisol at 1.8 ug/dL (normal range 4.8-19.5 ug/dL), and normal aldosterone and dehydroepiandrosterone sulfate (DHEA-S) levels. General surgery was consulted; given the size of the patient’s lesion and metastatic involvement, it was recommended that he be transferred to a tertiary care cancer center.

The patient subsequently arrived at our institution for further evaluation and management. A CT-guided biopsy was completed which surprisingly revealed metastatic melanoma (BRAF-negative). An MRI brain was completed to evaluate for intracranial metastasis; it returned within normal limits. The patient’s case was presented at a tumor board. Widespread metastatic disease made surgery a poor option for this patient, and an immunotherapy combination of ipilimumab and nivolumab was recommended. After completing one round of ipilimumab/nivolumab, a positron emission tomography (PET)/CT was completed; it revealed a pattern of large left adrenal malignancy with evidence of skeletal metastases and retroperitoneal nodes with additional involvement at the lateral right chest wall and posterior right upper abdominal wall. For continued difficulty with pain control from osseous metastasis, palliative radiation was completed with significant improvement in symptoms. Immunotherapy was also complicated by hyperglycemia necessitating hospitalization and changes to his home insulin regimen. Shortly after his third cycle, the patient presented to his local ED for abdominal pain. CT abdomen/pelvis showed significant disease progression with continued enlargement of the left adrenal mass as well as progressive hepatic and splenic metastatic lesions. He was treated with pain medications and discharged shortly afterward. His oncology team then switched him to nivolumab with relatlimab and added binimetinib, which he continues on at the time of this writing. 

## Discussion

Metastatic melanoma, an aggressive subtype of skin cancer, is notorious for its potential to disseminate across various organ systems. However, our case deviates from the more conventional metastatic pathways and resonates with an increasingly nuanced understanding of melanoma’s diverse presentations. 

While melanoma frequently metastasizes to the liver, lungs, brain, and bones, the combined manifestation involving the adrenal gland, liver, spleen, and bones is seldom observed [2]. Earlier studies have primarily focused on single-organ involvement, with few venturing into multifocal presentations [3,4]. Our case bridges these disparate accounts, portraying melanoma's potential to simultaneously invade multiple organs, notably the adrenal glands. Adrenal gland metastasis has consistently been linked to advanced disease stages and is frequently associated with a poor prognosis [5]. Therefore, when faced with adrenal metastases in a patient with melanoma, prompt diagnosis and timely therapeutic interventions become essential. 

The diagnostic trajectory our patient underwent was convoluted by the adrenal mass’s anomalous hormonal work-up. Elevated dopamine levels, along with normal epinephrine and norepinephrine levels, may easily misguide clinicians toward adrenal pathologies like pheochromocytoma, in which up to 40% of patients may have no symptoms [2,6]. If pheochromocytoma is on the differential, excluding it is important since a biopsy can precipitate a hypertensive crisis if there is an actively secreting tumor. Tests that can be used to help rule in or rule out such a diagnosis include 24-hour urine-fractionated metanephrines and catecholamines, as well as plasma-fractionated metanephrines [7]. Recent advances in imaging have also allowed laboratory testing to be substituted by CT in some cases [8]. Immunohistochemical staining of a biopsied tissue can be critical in differentiating between primary adrenal tumors and metastases to the adrenal glands. 

A biopsy from our patient ultimately confirmed the diagnosis of metastatic melanoma to the adrenal gland. Identifying and correctly characterizing a tumor informs treatment and timely intervention. An important feature of our case was the BRAF-negative status of the melanoma. Given that approximately 50% of melanomas harbor BRAF mutations [3], our patient's negative status eliminated the possibility of employing BRAF inhibitors, a common treatment for positive cases. Thus, our patient was treated with a programmed death receptor-1 (PD-1)-blocking antibody plus a lymphocyte activation gene-3 (LAG-3)-blocking antibody. The role of surgical intervention in the treatment of metastatic melanoma was also considered. One case report provided an example of a successful surgical resection of a patient’s metastatic melanoma to the adrenal gland, negative for BRAF mutation, whose primary melanoma site and subsequent lung metastases had already been resected years prior [9]. Another review concluded that for metastatic disease to the adrenal gland, an adrenalectomy should be performed when the metastasis is isolated to the adrenal gland and the primary tumor has been resected [7]. In our patient’s case, the widespread metastases involving the adrenal gland, bone, liver, and spleen made them a poor candidate for surgical resection. Multiple treatment options should still be explored and discussed within the interdisciplinary team and with the patient and should be pursued based on clinical history and findings. 

The subsequent development of hyperglycemia post-immunotherapy is another notable observation. Ipilimumab and nivolumab, immune checkpoint inhibitors, have revolutionized the treatment landscape of metastatic melanoma [4]. However, their potential to cause immune-related adverse events, including endocrinopathies, necessitates vigilant monitoring [2]. The observed hyperglycemia underscores this point and serves as a reminder of the delicate balance between efficacy and potential side effects as well as the necessity of careful monitoring for significant side effects. 

## Conclusions

This case report underscores the heterogeneity and aggressive nature of metastatic melanoma, particularly when it involves sites such as the adrenal gland. The concurrent involvement of the adrenal gland, liver, spleen, and bones in our patient highlights the unpredictable and multifaceted nature of melanoma metastases. The rapid progression of the adrenal lesion, despite its atypical hormonal profile, emphasizes the importance of a comprehensive diagnostic approach and the potential pitfalls of relying solely on hormonal work-ups. The BRAF-negative status of melanoma in our patient further accentuates the evolving landscape of therapeutic options, with immunotherapy emerging as a pivotal modality. In conclusion, this case reinforces the need for clinicians to maintain a high index of suspicion, employ a multifaceted diagnostic approach, and remain updated on the latest therapeutic modalities when managing patients with metastatic melanoma. 
